# Dimethylsulfoniopropionate Promotes Process Outgrowth in Neural Cells and Exerts Protective Effects against Tropodithietic Acid

**DOI:** 10.3390/md14050089

**Published:** 2016-05-06

**Authors:** Heidi Wichmann, Thorsten Brinkhoff, Meinhard Simon, Christiane Richter-Landsberg

**Affiliations:** 1 Aquatic Microbial Ecology Group, Institute for Chemistry and Biology of the Marine Environment (ICBM), University of Oldenburg, Oldenburg 26129, Germany; heidi.wichmann@uni-oldenburg.de (H.W.); thorsten.brinkhoff@icbm.de (T.B.); meinhard.simon@icbm.de (M.S.); 2 Molecular Neurobiology, Department of Neurosciences, University of Oldenburg, Oldenburg 26111, Germany

**Keywords:** nerve cells, oligodendrocytes, dimethylsulfoniopropionate, tropodithietic acid, neuroprotection, process outgrowth, *Roseobacter* clade bacteria

## Abstract

The marine environment harbors a plethora of bioactive substances, including drug candidates of potential value in the field of neuroscience. The present study was undertaken to investigate the effects of dimethylsulfoniopropionate (DMSP), produced by several algae, corals and higher plants, on cells of the mammalian nervous system, *i.e.*, neuronal N2a and OLN-93 cells as model system for nerve cells and glia, respectively. Additionally, the protective capabilities of DMSP were assessed in cells treated with tropodithietic acid (TDA), a marine metabolite produced by several *Roseobacter* clade bacteria. Both cell lines, N2a and OLN-93, have previously been shown to be a sensitive target for the action of TDA, and cytotoxic effects of TDA have been connected to the induction of oxidative stress. Our data shows that DMSP promotes process outgrowth and microtubule reorganization and bundling, accompanied by an increase in alpha-tubulin acetylation. Furthermore, DMSP was able to prevent the cytotoxic effects exerted by TDA, including the breakdown of the mitochondrial membrane potential, upregulation of heat shock protein Hsp32 and activation of the extracellular signal-regulated kinases 1/2 (ERK1/2). Our study points to the conclusion that DMSP provides an antioxidant defense, not only in algae but also in mammalian neural cells.

## 1. Introduction

Marine organisms produce a high diversity of structurally unique natural products with a broad spectrum of biological activities, including anti-cancer or antimicrobial effects, and some compounds even show a promising potential of neurotrophic or neuroprotective activity [1,2,3,4]. Natural products of marine algae, derived from the algae themselves or their bacterial associates, are a focal point in drug discovery programs due to their various biological activities [5,6]. In particular, it is of interest to establish their biomedical potential in the context of neurodegenerative diseases [1,5].

The algal compound dimethylsulfoniopropionate (DMSP) has multifunctional roles in the ocean, being a precursor for the climate relevant volatile dimethyl sulfide (DMS), acting as a substrate for marine bacteria such as *Roseobacter* [7,8], and as an important component of the marine sulfur cycle [9,10,11]. In algae, DMSP occurs at high concentrations and serves as osmolyte, cryoprotectant, and an antioxidant [9,12]. In addition to its role in natural biogeochemical processes, DMSP has also been found as a promising bioactive compound for biomedical research. It showed beneficial effects on stressed fish and crustaceans as well as diseased terrestrial animals, and has been demonstrated to exert beneficial effects on diseases such as breast cancer, induced diabetes and Parkinson [13,14]. Additionally, the use of algae as nutraceuticals, *i.e.*, foods and supplements that are advantageous to the health of consumers, is of interest, and in this respect beneficial effects of algal-based food may also be attributed to DMSP [13,14]. Furthermore, DMSP was shown to promote nerve growth factor (NGF) induced neurite outgrowth in rat pheochromocytoma PC12 cells and exerted protective effects against MPTP (1-methyl-4-phenyl-tetrahydropyridine) [15,16], a chemical that destroys dopaminergic neurons. Neurotrophins, such as NGF, are important for neuronal survival, development, differentiation, and functional maintenance of neurons in the central and peripheral system [17,18,19]. Hence, compounds with neurotrophin-like activities that promote cell survival and induce process outgrowth are promising therapeutic candidates in the treatment of neurodegenerative diseases [4,17], and may enable regenerative processes [20,21].

The aim of the present study was to investigate the effects of DMSP on mammalian neural cells. In particular, the question was addressed whether it affects cell morphology and process outgrowth and may have potential neuroprotective effects. Two cell lines were used, *i.e.*, mouse neuroblastoma N2a cells, as a model for neuronal cells [22], and rat oligodendroglial OLN-93 cells, which have been established as a model for the myelin forming cells of the central nervous system [23]. Cell morphology, cell viability and microtubule organization and posttranslational modification of tubulin were assessed. To study the neuroprotective potential of DMSP, cells were preincubated with DMSP and then treated with the marine secondary metabolite tropodithietic acid (TDA).TDA is a broad spectrum antibiotic produced by several bacteria affiliated with the genera *Phaeobacter*, such as *Phaeobacter inhibens* 17395 [24,25], *Ruegeria* and *Pseudovibrio*, all belonging to the marine *Roseobacter* group within the family of *Rhodobacteraceae* [26]. TDA is involved in the dynamic symbioses with microscopic algae [27], and inhibits a broad spectrum of both Gram-positive and -negative bacteria, including clinical pathogens, fungi, microalgae and promotes algal health by killing unwanted marine pathogens [24,27,28,29,30,31]. As we have shown before, TDA induced cytotoxic responses in both cell lines, caused the breakdown of the mitochondrial membrane potential, the activation of extracellular signal-regulated kinases ERK1/2 and the upregulation of the small heat shock protein HSP32, which has been linked to the induction of oxidative stress [32,33]. These effects were accompanied by disturbance of the microtubule network and an increase in the intracellular Ca^2+^-level [32]. These findings were recently supported by a study showing that due to its cytotoxic abilities TDA exhibited potent, broad-spectrum anticancer activities [31].

## 2. Results and Discussion

### 2.1. DMSP Induces Process Outgrowth, Microtubule Reorganization and Bundling

To investigate the effect of DMSP on process outgrowth, N2a and OLN-93 cells were treated with 1 mg/mL (7.4 mM) DMSP. We designed the experiments considering the concentrations of DMSP found in the natural environment, as it occurs together with tropodithietic acid (TDA) (see Conclusion). DMSP is amongst the most common metabolites in the marine environment and is produced by members of many marine algal phyla [11,34]. Here, some genera, families, or orders contain it in high concentrations (up to hundreds of mM) whereas within others only low concentrations are found (<10 mM) [11,34]. When it is released into the environment, DMSP is rapidly degraded by marine bacteria, such as roseobacters [8]. As determined by MTT (thiazolyl blue tetrazolium bromide) survival assay, DMSP did not exert cytotoxicity in both cell lines at concentrations up to 5 mg/mL (data not shown). Furthermore, high amounts of DMSP (30 mM) showed no adverse effects in rodents [14]. Thus, a concentration of 1 mg/mL (7.4 mM) was chosen in the present study. In comparison, other known antioxidants such as *N*-acetyl-l-cysteine, which is a precursor of glutathione, or the iron chelator deferoxamine also need to be applied in mM concentrations [35,36].

**Figure 1:**
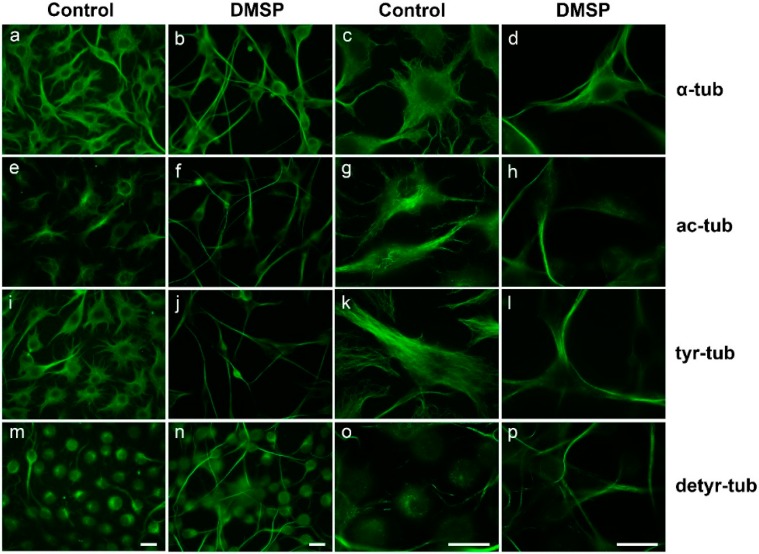
Effect of dimethylsulfoniopropionate (DMSP) on cell morphology in OLN-93 cells. OLN-93 cells were incubated with dimethyl sufoxide (DMSO) only (Control) or 1 mg/mL DMSP for 48 h. Indirect immunofluorescence staining was carried out with antibodies against α-tubulin (**a**–**d**); acetylated tubulin (**e**–**h**); tyrosinated tubulin (**i**–**l**) and detyrosinated tubulin (**m**–**p**) as indicated on the right. Magnification: left panel, 400×; right panel, 1000×. Scale bars: 20 µm.

**Figure 2:**
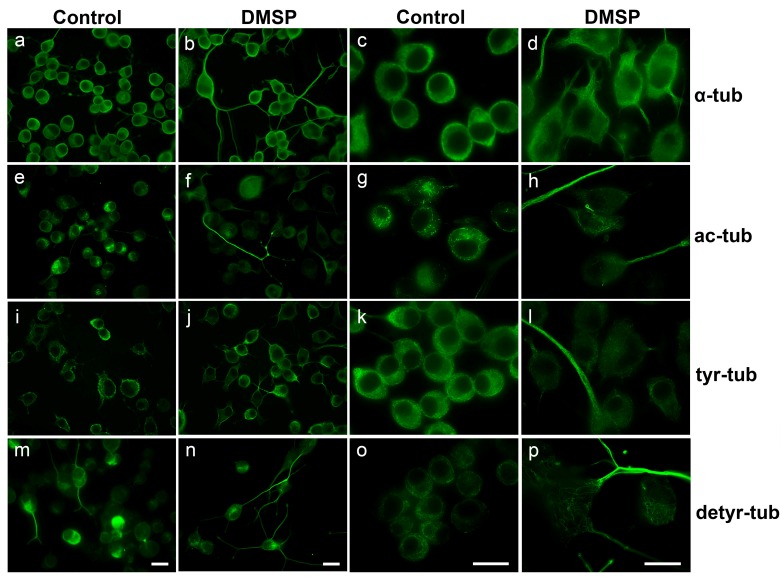
Effect of DMSP on cell morphology of N2a cells.N2a cells were incubated with DMSO only (Control) or 1 mg/mL DMSP for 48 h. Indirect immunofluorescence staining was carried out with antibodies against α-tubulin (**a**–**d**); acetylated tubulin (**e**–**h**); tyrosinated tubulin (**i**–**l**) and detyrosinated tubulin (**m**–**p**) as indicated on the right. Magnification: left panel, 400×; right panel, 1000×. Scale bars: 20 µm.

**Figure 3:**
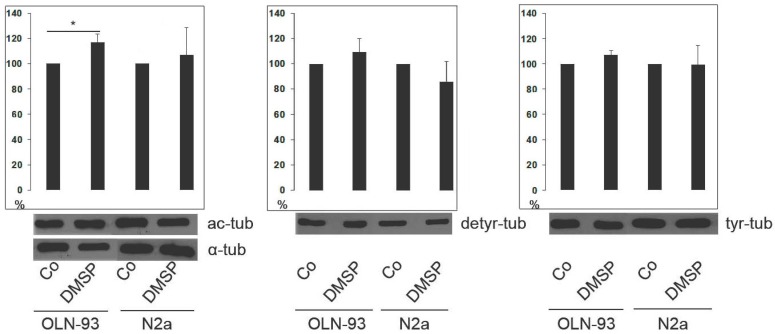
Effect of DMSP on tubulin acetylation and tyrosination. Cell lysates of OLN-93 and N2a cells were prepared and subjected to immunoblot analysis using antibodies as indicated on the right. Co, control cells; DMSP, cells were incubated with 1 mg/mL DMSP for 48 h. Quantitative evaluation of the immunoblots was carried out by densitometric scanning and Image Quant software (Molecular Dynamics, Sunnyvale, CA, USA). Acetylated tubulin (ac-tub), detyrosinated tubulin (detyr-tub) and tyrosinated tubulin (tyr-tub) is expressed as percentage of the total amount of α-tubulin, which was used as loading control (100%). Statistical evaluation was carried out by students *t*-test: * *p* < 0.05 significant.

Indirect immunofluorescence staining using antibodies against α-tubulin revealed that in both cell lines DMSP treatment caused morphological changes, and cellular processes containing a dense microtubule (MT) network appeared elongated (Figure 1 and Figure 2). Further, antibodies against acetylated tubulin (ac-tubulin) were used. Tubulin acetylation, at α-tubulin on Lys40, is considered as an indicator of MT stability [37]. After treatment with DMSP, ac-tubulin was specifically prominent in the long cellular extensions and hardly detectable in the cell soma (Figure 1 and Figure 2). Since tubulin acetylation is not strictly associated with stable (long-lived) MTs, we also determined the tyrosination state of tubulin by using antibodies against tyrosinated (tyr-tub) and detyrosinated tubulin (detyr-tub). Detyrosination of α-tubulin stabilizes indirectly MTs and is an indicator of enhanced MT stability, while dynamic MTs contain tyrosinated tubulin [37]. Figure 1 and Figure 2 demonstrate that DMSP led to the recruitment of both tyr-tub and detyr-tub to the cellular extensions. Immunoblot analysis of cell extracts revealed that in the presence of DMSP the amount of ac-tubulin was slightly but significantly enhanced, in OLN-93 cells, but not in N2a cells, while no significant changes in tyr- and detyr tubulin were observable in both cell lines (Figure 3).

Microtubules (MTs) are dynamically assembled polymers of α- and β-tubulin present in all eukaryotic cells. As constituents of the cytoskeleton, MTs are essential for various cellular processes, including mitosis, cell motility, intracellular transport, secretion, maintenance of cell shape and polarization [38]. MTs are heterogeneous in length and highly dynamic *in vivo* and *in vitro*, undergoing cycles of polymerization and rapid depolymerization. This “dynamic instability” is a feature that is crucial for many microtubule functions and modulated by interactions with other proteins, including microtubule motor proteins and non-motor microtubule-associated proteins (MAPs) [37]. As mentioned above, MT posttranslational modifications (PTMs), such as acetylation and detyrosination, are crucial for controlling MT stability and dynamics, as well as the interaction with other cellular components such as motor proteins and MAPs [39,40]. The present data indicate that DMSP alters the MT network and particularly causes the recruitment of rather stable MTs to the cellular processes, which may promote process formation and neurite outgrowth.

**Figure 4:**
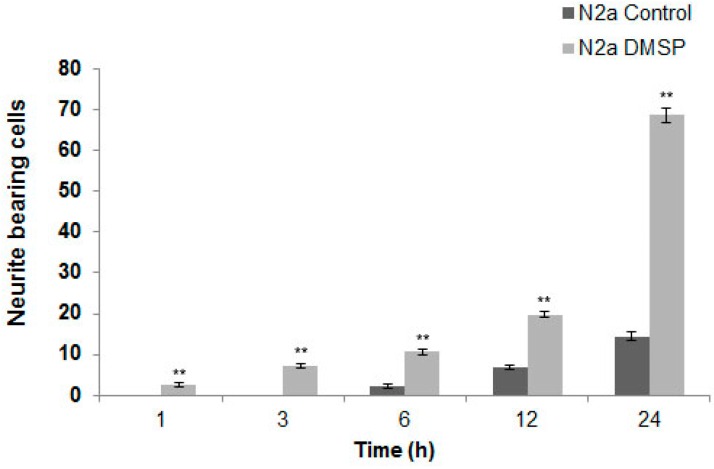
Effect of DMSP on process outgrowth in N2a cells. Cells were incubated with 1 mg/mL DMSP for the indicated times and process bearing cells were quantitatively evaluated. Statistical evaluation was carried out by students *t*-test: * *p* < 0.05 significant and ** *p* < 0.001 highly significant compared to the control.

As demonstrated in Figure 1 and Figure 2 DMSP induced process formation, cellular extensions appeared elongated and processes were prominently stained by antibodies against α-tubulin. To quantitatively evaluate this effect, N2a cells were used as an example, since these cells are more suitable to determine neurite outgrowth than OLN-93 cells, which have a more complex morphology. Cells with at least two cellular processes longer than two cell diameters were determined in control and DMSP (1 mg/mL; 24 h) treated cells. Figure 4 depicts that after the treatment with DMSP for 24 h a three-fold increase in neurite bearing cells was observed.

Taken together, the present data show that DMSP promotes reorganization of the MT network, which is accompanied by an enhanced acetylation of MTs, and process formation in neural cells, which similarly is observable in PC12 cells after the treatment with NGF [15,41,42].

### 2.2. DMSP Protects Neural Cells against Cytotoxic Effects Exerted by Tropodithietic Acid

**Figure 5:**
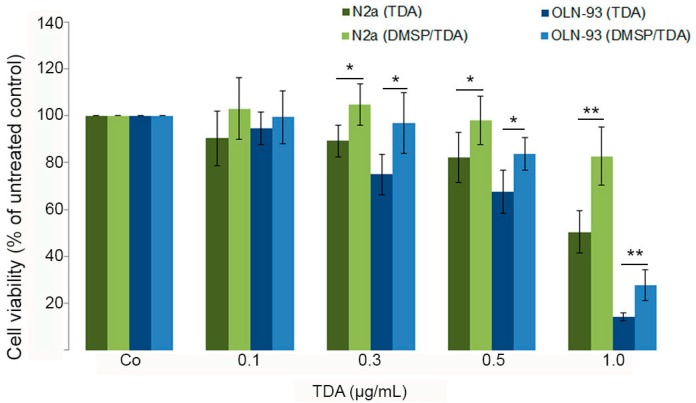
DMSP is protective against TDA induced cytotoxicity. OLN-93 and N2a cells were preincubated with DMSP (1 mg/mL, 24 h) followed by treatment with TDA for 24 h at the indicated concentrations and MTT (thiazolyl blue tetrazolium bromide) assay was carried out. Statistical evaluation was carried out by students *t*-test: * *p* < 0.05 significant and ** *p* < 0.001 highly significant compared to the control.

Our previous studies have shown that tropodithietic acid (TDA) induced morphological damages and cytotoxicity in OLN-93 and N2a cells. These effects were accompanied by activation of ERK1/2 and induction of heat shock protein 32 (HSP32). Furthermore, mitochondrial integrity was significantly impaired [32]. To assess whether DMSP is capable to protect cells against TDA induced damages, OLN-93 and N2a cells were preincubated with DMSP (1 mg/mL) for 24 h followed by the treatment with TDA for 24 h as indicated in the presence of DMSP. To quantitatively determine cytotoxic effects, an MTT was carried out. Figure 5 indicates that preincubation with DMSP exerts protective effects and cells display an approximately 20% higher survival rate after the treatment with TDA (0.3–0.5 µg/mL). This effect was even more pronounced after the treatment with 1 µg/mL TDA, however, in particular OLN-93 cells were severely damaged and only about 30% of the cells could be rescued under these conditions (Figure 5).

### 2.3. DMSP Suppresses TDA Induced Stress Responses and Mitochondrial Damage

**Figure 6:**
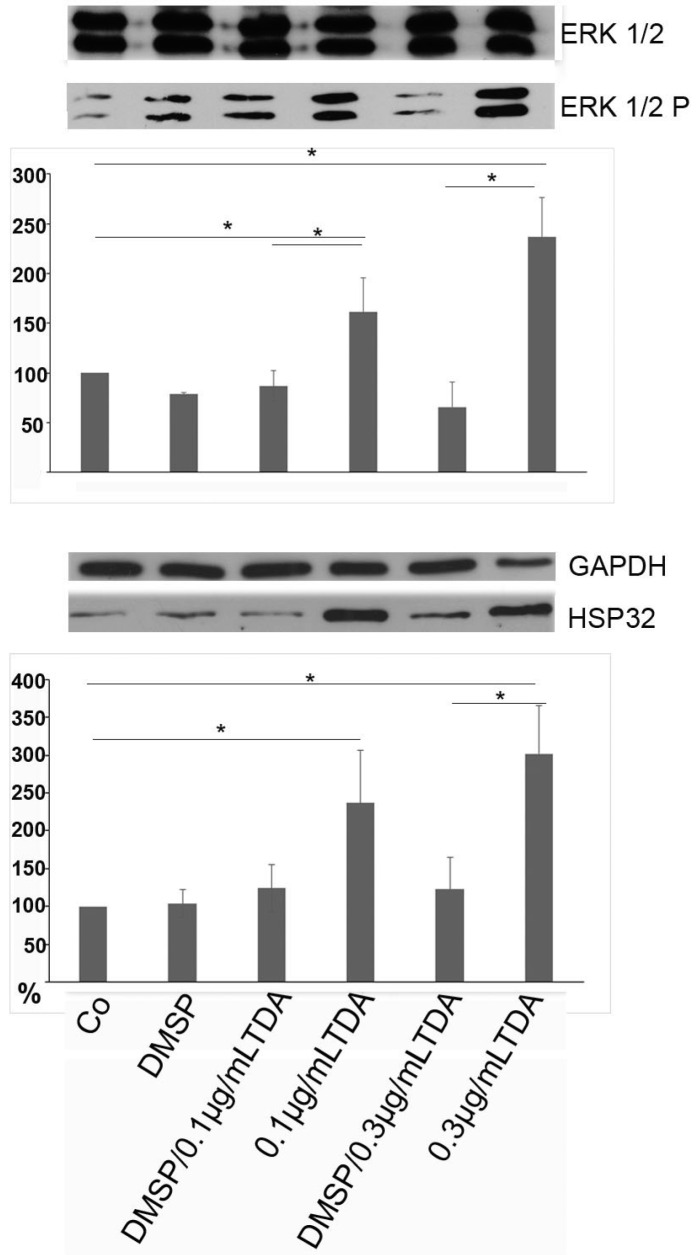
Protective effect of DMSP against TDA induced stress responses. Cell lysates of OLN-93 cells were prepared and subjected to immunoblot analysis using antibodies as indicated on the right. DMSP, preincubation with 1 mg/mL DMSP for 24 h; DMSP/TDA, preincubation with 1 mg/mL DMSP for 24 h and subsequent exposure to TDA for 24 h in the presence of DMSP. Co, untreated control. Quantitative evaluation of the immunoblots was carried out by densitometric scanning and Image Quant software (Molecular Dynamics, Sunnyvale, CA, USA). Activated ERK1/2 (ERK1/2 P) is expressed as percentage of the total amount of ERK1/2 (100%). HSP32 is expressed as percentage of glyceralhyde 3-phosphate dehydrogenase (GAPDH, 100%). Statistical evaluation was carried out by students *t*-test: * *p* < 0.05 significant compared to the control or the TDA treated samples without DMSP preincubation.

In the following only OLN-93 cells were used, since similar effects were observed in both cell lines, and these cells were more sensitive to the effects of TDA. As mentioned above, activation of extracellular signal-regulated kinases 1 and 2 (ERK1/2) and the induction of HSP32 underlie the cytotoxic effects of TDA [32]. To investigate whether DMSP can suppress these responses, cells were preincubated with DMSP (1 mg/mL, 24 h) followed by the treatment with TDA (0.1 or 0.3 µg/mL) as indicated. Cell lysates were prepared and immunoblot analysis using antibodies against total ERK1/2, HSP32 and GAPDH (glyceraldehyde 3-phosphate dehydrogenase) as a loading control was carried out. Figure 6 reveals that in presence of DMSP the TDA-induced ERK1/2 activation (upper panel) and induction of HSP32 (lower panel) was significantly reduced. In comparison to the treatment with TDA (0.3 µg/mL) alone, activated ERK1/2 was reduced approximately fourfold from 240% to 60%, and HSP32 threefold from 250% to 80%.

Next, we assessed whether DMSP can protect cells from mitochondrial damage exerted by TDA. To monitor mitochondrial morphological damage and functional properties, live OLN-93 cells were stained with MitoTracker Red, which accumulates in living cells and is used to assess the integrity of the mitochondrial membrane potential. Additionally, indirect immunofluorescence using antibodies against heat shock protein 60 (Hsp60) was carried out. Hsp60 associates with the mitochondrial matrix, where it participates in the folding and assembly of transported proteins, and can be used to investigate mitochondrial distribution and integrity after mitochondrial damage. It may be released and then appear rather diffusely distributed throughout the cell soma. Hsp60 has been suggested to play a role in pro-survival or pro-apoptotic pathways [43,44].

**Figure 7:**
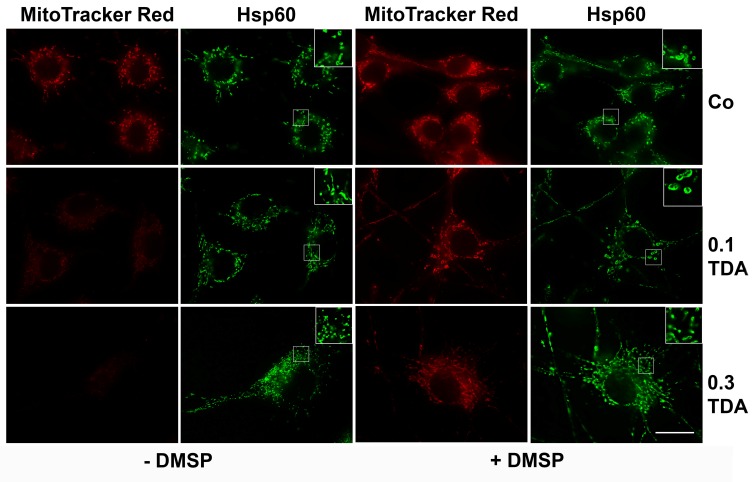
Protective effect of DMSP against TDA-induced mitochondrial damage in OLN-93 cells. Cells were incubated with DMSO only (Co, −DMSP) or preincubated with 1mg/mL DMSP for 24 h (Co, +DMSP), followed by incubation with 0.1 or 0.3 µg/mL TDA for 24 h in the presence of DMSP. Afterwards, live cells were stained for 30 min with MitoTracker Red, fixed with methanol and subjected to indirect immunofluorescence using antibodies against HSP60. Magnification: 1000×. Scale bar 20 µm.

Figure 7 shows that in control cells, mitochondria are evenly distributed throughout the cell soma. Similar to our previous study [32], treatment with TDA (0.1 or 0.3 µg/mL, 24 h) caused a breakdown of the mitochondrial membrane potential, indicated by disappearance of the red fluorescent MitotrackerRed signal. Hsp60 staining showed their association with the mitochondria and no release into the cytoplasm. Additionally, the mitochondria appeared smaller (Figure 7). After preincubation with DMSP (1 mg/mL, 24 h) mitochondria in TDA treated cells were protected, the membrane potential was preserved and Hsp60 distribution was similar to the untreated control cells. Hence, DMSP was capable to exert a protective role against TDA induced mitochondrial impairment.

Taken together, DMSP is protective against TDA induced cytotoxicity in mammalian cells of the nervous system. As shown in our previous study, TDA causes oxidative stress indicated by upregulation of HSP32 and mitochondrial damage [32]. Pretreatment with the antioxidant DMSP combined with subsequent exposure to TDA suppressed the depolarization of Δψ_m_. Additionally, the induction of stress induced protein Hsp32/HO1 and activation of ERK1/2 did not occur. Hence, DMSP was capable to prevent the stress responses exerted by TDA.

Other compounds derived from algae have been demonstrated to exert antioxidative effects. In this respect, the best investigated beneficial compounds are docosahexaenoic acid (DHA) and eicosapentaenoic acid (EPA) [45]. They have antioxidative properties, stimulate neural development, promote neurogenesis and accumulate as the most abundant fatty acids in the brain [46,47]. The beneficial effects of these compounds are receiving more and more attention in particular regarding their antioxidative abilities. They are discussed especially in context of the nutraceutical potential of algae, as a growing body of evidence suggests that nutrition plays an important role in the development of neurodegenerative diseases [48]. For instance, patients with Alzheimer disease (AD) display a bad nutritional status, which was reported to enhance disease progression [49]. Many studies support the important role of antioxidants in the prevention of AD as this disease is accompanied by the occurrence of oxidative stress [49,50]. Furthermore, it was shown that DHA plays an important role in the prevention of neuropsychiatric and neurodegenerative disorders [51].

Altogether, algae are considered as a rich source of natural antioxidants due to the presence of various secondary metabolites with antioxidative effects [52,53]. The potential role of DMSP in this context is rarely understood and the protective activity of DMSP is of special interest regarding the use of algae as nutraceuticals. Our findings suggest that DMSP does not only work as an efficient protective and antioxidative system in algae [12], but also in mammalian neural cells. Hence, it might be speculated that the beneficial effects of algal-based food could also be attributed DMSP. However, further investigations are needed to elucidate the nutraceutical potential of DMSP and its underlying molecular mechanisms.

## 3. Experimental Section

### 3.1. Materials and Antibodies

Cell culture media were purchased from Gibco/BRL (Grand Island, NY, USA). Poly-l-lysine (PLL), from Sigma (Munich, Germany). Dimethylsulfoniopropionate (DMSP) and tropodithietic acid (TDA) were purchased from Bioviotica Naturstoffe GmbH (37077 Göttingen, Germany), dissolved in DMSO and stored in dark at −20 °C. DMSO was always included in control experiments.

For Western blot analysis, the following antibodies were used, working dilutions are given in brackets: mouse monoclonal antibody (mAb) anti-α-tubulin (1:1000), and mouse mAb anti acetylated α-tubulin (1:1000), anti-extracellular regulated kinase 1/2 (ERK1/2, 1:2000) and mouse mAb ERK1/2-P (1:1000) from Sigma (Munich, Germany), mouse mAb anti-GAPDH (1:1000) from Sigma-Aldrich (St. Louis, MO, USA). Monoclonal antibody anti-HSP32 1:1000) from Enzo Lifesciences (Lörrach, Germany). Rabbit polyclonal antibody (pAb) anti detyrosinated α-tubulin (1:1000) was obtained from Merck Millipore (Darmstadt, Germany) and rat mAb anti tyrosinated α-tubulin clone YL1/2 (1:1000) from Santa Cruz (Dallas, TX, USA).

For immunochemistry, the following antibodies were used, working dilutions are given in brackets: mouse mAb anti-α-tubulin (1:250), mouse mAb anti-acetylated α-tubulin (Lys40) (1:250), rat mAb anti-tyrosinated α-tubulin clone YL1/2 (1:200) and rabbit pAb anti-detyrosinated α-tubulin (Glu-tubulin) (1:200) from Millipore, Billerica, MA, USA. Mouse mAb anti-HSP60 (1:1000) was from Enzo Lifesciences. The antibody Glu-tubulin specifically recognizes the detyrosinated form of α-tubulin and binds at the *C*-terminus (Glu tubulin). Ab YL1/2 is specific for detection of the tyrosinated form of α-tubulin. Acetylation of Lys40 is localized at the amino‑terminal domain of α-tubulin, and the monoclonal anti-acetylated α-tubulin antibody recognizes an epitope of Lys40, within four residues, when this amino acid is acetylated.

### 3.2. Cell Culture

In this study, OLN-93 cells, an oligodendroglial cell line derived from rat brain glial cultures [23] and N2a (wt) cells, a mouse derived neuroblastoma cell line [22], were used. Cells were kept in Dulbecco’s modified Eagle medium (DMEM) supplemented with 10% heat inactivated fetal bovine serum (FBS) for OLN-93 cells and 0.5% FBS for N2a cells, 2 mM Glutamine, 50 U/mL penicillin (P), and 50 μg/mL streptomycin (S) at 37 °C and 10% CO_2_ [23]. In all subsequent experiments, DMSO was added to control cultures. All experiments were carried out at least three times with similar results. Cells were monitored by Hoffman modulation contrast microscopy.

### 3.3. Immunoblot Analysis

Cellular monolayers of control and treated cells were washed once with PBS, scraped off in sample buffer containing 1% SDS, and boiled for 10 min. The protein contents were determined according to Neuhoff *et al.* [54]. For immunoblotting, total cellular extracts (10–20 μg protein per lane) were separated by one-dimensional SDS polyacrylamide gel electrophoresis (SDS-PAGE) using 8.75%–10% polyacrylamide gels and blotted onto nitrocellulose membranes (Whatman, Dassel, Germany; 0.2 μm). The blots were saturated with TBS (20 mM Tris, 136.8 mM NaCl, pH 7.5) containing 5% dry milk and incubated with the individual antibodies overnight at 4 °C. After washing with Tris-buffered saline (TBS) with 0.1%·v/v Tween 20 (TBS-T), blots were incubated with HRP-conjugated anti-mouse (1:10,000) or anti-rabbit (1:10,000) antibodies for 1 h at RT. After washing with TBS-T, blots were visualized by the enhanced chemiluminescence procedure as described by the manufacturer (Thermo Scientific, Rockford, IL, USA). All experiments were carried out at least three times with similar results.

### 3.4. Mitochondrial Staining

OLN-93 cells (3.5 × 10^5^ cells/10 cm dish) were cultured on PLL-coated glass cover slips for 24 h in DMEM/10% FBS and subjected to treatment as indicated. N2a cells (1.2 × 10^6^ cells/10 cm dish) were cultured on HCl treated glass coverslips without PLL for 24 h in DMEM/0.5% FBS and incubated with MitoTracker Red (100 nM) (Molecular Probes, Oregon, OR, USA) for 30 min, washed twice with PBS and fixed with ice-cold methanol for 7 min or with 3% paraformaldehyde. The latter were permeabilized with 0.1% Triton X-100. Thereafter, indirect immunofluorescence staining was carried out as described below.

### 3.5. Indirect Immunofluorescence

OLN-93 cells (3.5 × 10^5^ cells/10 cm dish) were cultured on PLL-coated glass coverslips for 24 h in DMEM/10% FBS and subjected to treatment as indicated. N2a cells (1.2 × 10^6^ cells/10 cm dish) were cultured on HCl treated glass coverslips without PLL for 24 h in DMEM/0.5% FBS. Cells were incubated overnight at 4 °C with the indicated antibodies. After washing with PBS, cells were incubated for 1h with Dylight (488 as well as 594) conjugated (1:400; Thermoscientific, Rockford, IL, USA) and FITC-conjugated (1:100) secondary antibodies (Santa Cruz Biotechnology Inc., Heidelberg, Germany) washed with PBS and mounted. Nuclei were stained by 4C, 6-diamidino-2-phenylindole (DAPI) (1.5 mg/mL) included in the mounting medium (Vectashield; Vector Laboratories, Burlingame, CA, USA). Fluorescent labeling was studied using a Zeiss epifluorescence microscope (Oberkochen, Germany) equipped with a digital camera using a planneofluar objective (40× magnification for overview images, 100× magnification for detailed images).

### 3.6. MTT-Viability Assay

To assess the protective effect of DMSP and cytotoxic potential of TDA the MTT (tetrazolium) assay was carried out as described before [35]. Briefly, OLN93 or N2a cells were prepared as described above, plated on (OLN-93 cells; PLL-coated) 96-microwell cell culture plates (3500 or 12,000 cells per well) and incubated for 24 h. The growth medium was removed and fresh medium (100 µl/well) was added, some cells were preincubated with 1 mg/mL DMSP for 24 h. Growth medium was removed and fresh medium was added and subsequently 1 mg/mL DMSP was added for additional 24 h. Subsequently, 0.1 µg/mL–1 µg/mL TDA was added for additional 24 h. 10 µl of MTT solution (5 mg/mL in PBS) were added to the wells (each containing 100 µL medium) and the plates were incubated for 2 h. 100 µL of a solubilization solution (10% sodium dodecyl sulfate in 0.01 mol/L HCl) was added and incubated overnight to dissolve the water-insoluble formazan salt. Quantification was carried out with an ELISA reader at 595 nm using a 655-nm filter as a reference. Data are expressed as percentage of the untreated controls, with each value representing the mean ^ SD of eight microwells from three independent experiments (*n* = 24).

## 4. Conclusions

Marine compounds have been demonstrated to modulate signaling pathways that are involved in the regulation of cell death and survival [5]. Since neurodegenerative diseases are connected to a variety of stress situations, including nitrative and oxidative damage, the development of drugs with antioxidant activity is highly important. Low molecular weight compounds, which mimic the activity of neurotrophins and are capable to cross the blood brain barrier (BBB), are promising therapeutics [4,17]. Our data indicate that DMSP exerts antioxidative effects, and previously was demonstrated to have neuroprotective abilities in rodents [14]. Its closely related compound dimethyl sulfone (DMSO_2_) is able to cross the highly selective blood brain barrier (BBB) [55], which implicates that DMSP similarly may be able to cross the BBB. Thus, in combination with its effects on the microtubule network and outgrowth promoting capability DMSP displays an interesting biomedical potential, and this demands further investigations.

Furthermore, our findings raise questions regarding a putative chemical-ecological role of DMSP and TDA in the natural marine environment. We have shown that DMSP appears to confer an antioxidative effect in the used cell models, as TDA potentially induces oxidative stress [32]. Although algae cannot be directly compared with neural cells, the main molecular mechanisms of eukaryotic cells are similar and thus this might be comparable to the natural effects in algae. DMSP producing marine microalgae such as *Emiliania huxleyi*, and the TDA producing bacterium *Phaeobacter inhibens*, were found in a symbiotic relationship, building an algal-bacterial mini-ecosystem [27,31]. *P. inhibens* and other organisms of the *Roseobacter* clade are supposed to stimulate algal growth by biosynthesizing and secreting antibiotics against pathogenic bacteria such as TDA, and growth factors [27]. In return, some roseobacters including *P. inhibens* can use DMSP as a carbon and sulfur source [27]. TDA, however, was also found to inhibit the growth of some microalgae [26,56]. Considering our results regarding the protective effect of DMSP against TDA induced toxicity, the present study suggests that DMSP might also play a protective role in the interaction between microbes and their algal hosts.
